# Traumatic Mitral Valve and Pericardial Injury

**DOI:** 10.1155/2013/385670

**Published:** 2013-09-10

**Authors:** Nissar Shaikh, Firdous Ummunissa, Mohamed Abdel Sattar

**Affiliations:** ^1^Surgical Intensive Care Unit, Department of Anesthesia/ICU, Hamad Medical Corporation, P.O. Box 3050, Doha, Qatar; ^2^Department of Medical Education, Hamad Medical Corporation, P.O. Box 3050, Doha, Qatar; ^3^Department of Cardiology and Cardiothoracic Surgery, Hamad Medical Corporation, P.O. Box 3050, Doha, Qatar

## Abstract

Cardiac injury after blunt trauma is common but underreported. Common cardiac trauma after the blunt chest injury (BCI) is cardiac contusion; it is very rare to have cardiac valve injury. The mitral valve injury during chest trauma occurs when extreme pressure is applied at early systole during the isovolumic contraction between the closure of the mitral valve and the opening of the aortic valve. Traumatic mitral valve injury can involve valve leaflet, chordae tendineae, or papillary muscles. For the diagnosis of mitral valve injury, a high index of suspicion is required, as in polytrauma patients, other obvious severe injuries will divert the attention of the treating physician. Clinical picture of patients with mitral valve injury may vary from none to cardiogenic shock. The echocardiogram is the main diagnostic modality of mitral valve injuries. Patient's clinical condition will dictate the timing and type of surgery or medical therapy. We report a case of mitral valve and pericardial injury in a polytrauma patient, successfully treated in our intensive care unit.

## 1. Background 

Traumatic cardiac injury is one of the common unsuspected organ injuries leading to fatal outcome in polytrauma patients [1]. Cardiac injuries following blunt chest trauma vary from cardiac contusions to the fatal myocardial rapture. The cardiac injury occurs in up to 76% of the blunt chest trauma patients, but trauma to cardiac valves is a rare finding [1]. Rupture of papillary muscle or its tendinous cords following blunt trauma is a very rare etiology of the acute mitral regurgitation. We report a case of mitral valve and pericardial injury in a polytrauma patient, successfully treated in our intensive care unit.

## 2. Illustrated Case

**Figure 1:**
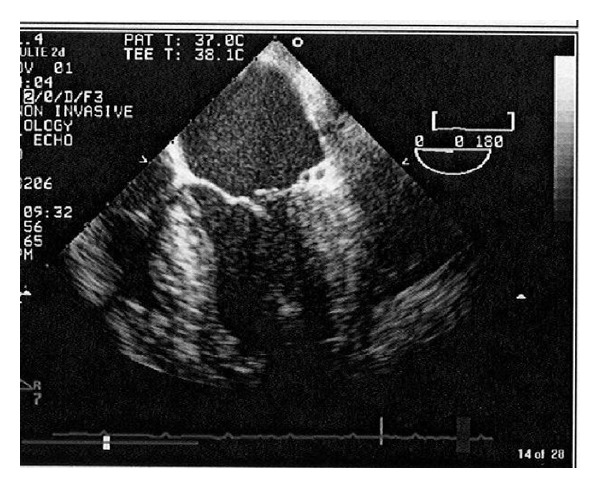
Raptured cordae tendineae.

**Figure 2:**
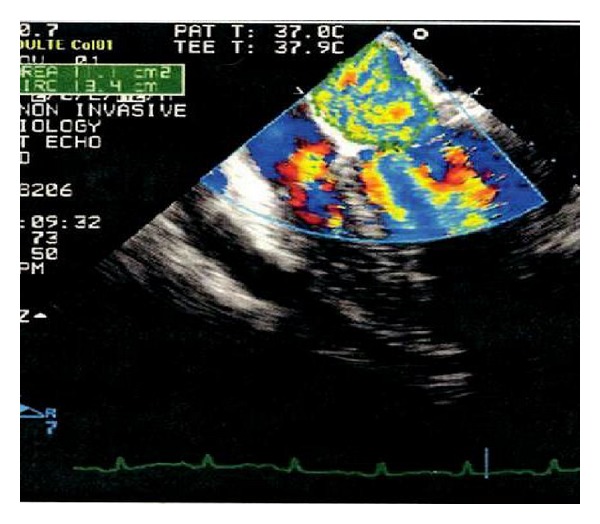
Severe mitral regurgitation.

A 48 years old pedestrian was involved in a road traffic accident, and on admission, the Glasgow coma score (GCS) was 14/15, tachypneic (respiratory rate was 35 to 40/minute), tachycardic (heart rate was 120 to 140/minute), with systolic blood pressure 90 mm of Hg and oxygen saturation of 88% to 90% on 15 litres/minute of oxygen supplementation. He had a flail chest with decreased air entry on left side. Chest X-ray showed fractured ribs (1 to 7) on left side with hemopneumothorax. Immediate left chest drain was inserted. Initial computerized tomography (CT) scans reveled bilateral lung contusions with fractured ribs on left side. He had grade IV splenic injury and hemoperitoneum. An immediate laparotomy showed ruptured left diaphragm and mesenteric venous bleeding. Splenectomy with diaphragm repair and ligation of mesenteric vein was done. Patient was shifted postoperatively to the intensive care unit. He remained intubated and ventilated, and resuscitation was continued with blood and blood products. Antiulcer medication and antibiotics were added to the therapy. He was having tachycardia (120 to 140 beats/min), inspite of good sedation and analgesia guided by bispectral index (BIS) monitoring. On systemic examination, he was found to have new pansystolic murmur. Electrocardiogram (ECG) showed sinus tachycardia and multiple ectopic beats. Echocardiography (Echo) revealed flail posterior mitral valve, ruptured chordae tendineae (Figure 1), and severe mitral regurgitation (Figure 2) with good left ventricular function (ejection fraction 60%) and mild to moderate pericardial effusion. Patient was started on angiotensin converting enzymes (ACE) inhibitors. His hemodynamics improved, but despite of aggressive physiotherapy and positioning, X-ray on chest showed left basal collapse consolidation. On day 4, bronchoalveolar lavage was done but no significant improvement of the collapse segment happened. Chest CT scan confirmed left lung basal collapse consolidation and severe pericardial effusion with the pressure effects on left lung leading to basal collapse consolidation. There was no cardiac tamponade. As it was difficult to wean him from the ventilator, tracheostomy was done on day 6. Echo was repeated and showed good left ventricular ejection fraction but severe pericardial effusion (Figure 2). He underwent thoracotomy and drainage of the pericardial effusion on day 8. It drained 820 mL of serosanguineous fluid in 24 hours. By day 12, his chest condition improved, and the left lung basal collapse consolidation was resolved. He was started to wean from the ventilator, but, on day 16, developed pulmonary edema, and pulmonary artery catheter was inserted. Pulmonary artery wedge pressure was high (22 mm of Hg), he was started on frusemide and digoxin. His clinical condition improved, and he was able to breathe spontaneously by day 21, his trachea was decannulated on day 22, he was transferred to ward on day 23, and he was subsequently discharged to be followed in outpatient department. 

## 3. Discussion

Cardiac injury after blunt trauma is common but underreported [1]. Common cardiac trauma after the blunt chest injury (BCI) is cardiac contusion; cardiac valve injury is very rare. When the cardiac valve trauma occurs, it commonly involves the aortic valve then the mitral and tricuspid valve [2]. Rarity of the mitral valve trauma is reflected from the review of two studies. Parmley et al. [3] did not find a single case of isolated mitral valve injury in autopsy of 546 patients with cardiac injuries. Maenza et al. [4] in their meta-analysis of the blunt cardiac trauma did not mention a single case of isolated cardiac valve injury. Mitral regurgitation due to ischemia and other etiologies affects more than 2 million people in the United States [5], but acute mitral regurgitation due to mitral valve injury is reported in only 82 cases in the literature [6]. The mitral valve injury in chest trauma occurs when extreme pressure is applied at early systole during the isovolumic contraction between the closure of the mitral valve and the opening of the aortic valve [7]. Experimental studies have shown that an intraventricular pressure greater than 320 mm of Hg is needed to cause any cardiac wall or valve rupture [8].

Traumatic mitral valve injury can involve valve leaflet, chordae tendineae, or papillary muscles. The posterior leaflet has much thinner chordae than the anterior ones, and these thinner chordae are weaker in comparison to anterior leaflet chordae and are more at the risk of rupture in blunt trauma [9]. The papillary muscles are supplied by long penetrating arteries arising from the epicardial vessels and if they are injured, the muscle and chordae undergo progressive ischemia, subsequent infarction, and paresis leading to delayed rupture [10]. For the diagnosis of mitral valve injury, a high index of suspicion is required, as in polytrauma patients, other obvious severe injuries will divert the attention of the treating physician. Clinical picture of patients with mitral valve injury may be none to cardiogenic shock. Commonly, these patients will be having tachycardia and new pansystolic murmurs which radiate to the base of the heart. Chest X-ray may show cardiomegaly. Electrocardiogram (Echo) frequently reveals sinus tachycardia. The echocardiogram is the main diagnostic modality of mitral valve injuries. The transthoracic echocardiogram (TTE) has a limited role in polytrauma patients, as there will be a poor window due to dressing, interference from pneumothorax, and inability to give proper position to the patient. The transesophageal echocardiogram (TEE) has higher resolution sonography and is not affected by the previously mentioned factors [11]. If the injury to the papillary muscle is complete and involves anterior papillary muscle, the clinical picture is acute with the hemodynamic instability and most of these patients will require emergency surgical intervention [6]. Traumatic mitral insufficiency if not detected early and treated properly can get complicated and progress to congestive heart failure and cardiogenic shock. Patient's clinical condition will detect the timing and type of surgery or medical therapy. When deciding for surgery, it is of vital importance to consider local valve injury, the associated injuries, and timing of injury and its detection [12]. Fifty-seven percent of total reported cases required a valve replacement surgery [6]. Mitral valve annuloplasty with the pericardial strip is another way of conservative surgery for mitral valve injury; it preserves the anatomy and physiological function of the valve annulus and provides better long-term results [13].

Shammas et al. reported treating the traumatic mitral regurgitation successfully by the medical therapy [1]. Our patient also was managed medically as he had good left ventricular function and risk of anticoagulation with the associated injuries.

Huang et al. reported that 87% of their patients with pericardial effusion were the result of blunt chest trauma and required surgical drainage [14]. In our patient, the pericardial effusion with clotted blood was causing a clinically significant pressure effect on the left lung; hence surgical drainage was done.
